# Psychosocial determinants for adherence to a healthy lifestyle and intervention participation in the FINGER trial: an exploratory analysis of a randomised clinical trial

**DOI:** 10.1007/s40520-022-02088-x

**Published:** 2022-02-19

**Authors:** Elisa Neuvonen, Jenni Lehtisalo, Alina Solomon, Riitta Antikainen, Satu Havulinna, Tuomo Hänninen, Tiina Laatikainen, Jaana Lindström, Nina Rautio, Hilkka Soininen, Timo Strandberg, Jaakko Tuomilehto, Miia Kivipelto, Tiia Ngandu

**Affiliations:** 1 School of Medicine, Institute of Clinical Medicine/Neurology, University of Eastern Finland, P.O. Box 1627, 70211 Kuopio, Finland; 2 Population Health Unit, Finnish Institute for Health and Welfare, P.O. Box 30, 00271 Helsinki, Finland; 3 Division of Clinical Geriatrics, Center for Alzheimer Research, Department of Neurobiology, Care Sciences, and Society, Karolinska Institutet, Solnavägen 1, 171 77 Solna, Sweden; 4 Ageing Epidemiology Research Unit, School of Public Health, Imperial College London, St Dunstan’s Road, London, W6 8RP UK; 5 Center for Life Course Health Research/Geriatrics, University of Oulu, P.O. Box 5000, 90014 Oulu, Finland; 6 Medical Research Center Oulu, Oulu University Hospital, P.O. Box 10, 90029 OYS Oulu, Finland; 7 Ageing, Disability and Functioning Unit, Finnish Institute for Health and Welfare, P.O. Box 30, 00271 Helsinki, Finland; 8 Department of Neurology, Neurocenter, Kuopio University Hospital, P.O. Box 100, 70029 KYS Kuopio, Finland; 9 Institute of Public Health and Clinical Nutrition, University of Eastern Finland, P.O. Box 1627, 70211 Kuopio, Finland; 10 Joint Municipal Authority for North Karelia Social and Health Services (Siun Sote), Tikkamäentie 16, 80210 Joensuu, Finland; 11 University of Helsinki and Helsinki University Hospital, P.O. Box 340, 00029 HUS Helsinki, Finland; 12 Department of Public Health, University of Helsinki, P.O. Box 20, 00014 Helsinki, Finland; 13 South Ostrobothnia Central Hospital, Hanneksenrinne 7, 60220 Seinäjoki, Finland; 14 National School of Public Health, Carlos III Health Institute, Monforte de Lemos 5, 28029 Madrid, Spain; 15 Diabetes Research Group, King Abdulaziz University, P.O. Box 80215, Jiddah, 21589 Saudi Arabia; 16 Theme Aging, Karolinska University Hospital, Karolinska Vägen 22, 171 64 Solna, Sweden

**Keywords:** Adherence, Clinical trial, Dementia prevention, Lifestyle, Older adults, Participation, Psychosocial factors

## Abstract

**Background and aims:**

Psychosocial factors may affect adherence to lifestyle interventions and lifestyle changes. The role of psychosocial factors in dementia prevention needs more research. We aimed at clarify the issue in the Finnish Geriatric Intervention Study to Prevent Cognitive Impairment and Disability (FINGER).

**Methods:**

The population included 1260 participants aged 60–77 years at risk for cognitive decline, randomised to a multidomain lifestyle intervention or regular health advice for 2 years. Adherence was evaluated as participation in the provided activities and actual lifestyle changes, separately for each domain (diet, exercise, social/cognitive activity, vascular risk management) and combined into multidomain. Psychosocial factors were measured at trial baseline (depressive symptoms; study perception; health-related quality of life, *HRQoL*) and earlier life (hopelessness; satisfaction with family life, achievements, and financial situation).

**Results:**

Depressive symptoms, hopelessness, and nonpositive study perception were negatively and *HRQoL* positively associated with participation in the multidomain intervention. Depressive symptoms, lower *HRQoL*, hopelessness and dissatisfaction with financial situation were associated with unhealthier lifestyles at baseline. Baseline depressive symptoms and lower *HRQoL* predicted less improvement in lifestyle, but did not modify the intervention effect on lifestyle change.

**Discussion and conclusions:**

Several psychosocial factors were associated with participation in lifestyle intervention, while fewer of them contributed to lifestyle changes. Although the intervention was beneficial for lifestyle changes independent of psychosocial factors, those most in need of lifestyle improvement were less likely to be active. Tailoring lifestyle-modifying strategies based on the need for psychosocial support may add efficacy in future trials.

**Trial Registry:**

ClinicalTrials.gov NCT01041989 2010-01-05

**Supplementary Information:**

The online version contains supplementary material available at 10.1007/s40520-022-02088-x.

## Background

Cognitive impairment and dementia affect a large number of ageing individuals. Currently, around 50 million people worldwide are living with dementia, and this number is estimated to triple by 2050 [1]. Alzheimer’s disease, the most common cause of dementia, is a multifactorial disease with several established potentially modifiable risk factors [1]. Accordingly, dementia prevention strategies have shifted towards multimodal lifestyle-based approaches [2]. Psychosocial factors, such as depression and hopelessness, are known to increase the risk of dementia in observational studies [3, 4], but their role in dementia prevention is largely unclear. Psychosocial predictors for a lifestyle change or participation in lifestyle interventions have been studied rarely overall or in multimodal interventions in the dementia prevention context.

Multiple factors including individual characteristics, type and intensity of intervention, and delivery methods are important in determining participation in intervention trials [5]. Among psychosocial factors, depressive symptoms have been quite consistently associated with poorer participation in the lifestyle interventions [5–8], or with greater dropout [9], but they have not always hampered reaching beneficial lifestyle modifications [10]. Positive attitude and self-efficacy have been associated with higher adherence to exercise programmes [6, 11, 12].

Quality of life and life satisfaction have perhaps been more often studied as consequences than predictors of lifestyle, but some studies have evaluated their role in the lifestyle change or participation. Lower self-rated health and low health-related quality of life (*HRQoL*) have predicted poorer participation in physical exercise [7, 11], and quality of life has been identified as a lifestyle change facilitator [13].

Depressive symptoms have been consistently associated with poorer adherence to healthy lifestyle habits in observational studies, e.g. exercise or diet [14, 15], and also motivation and stress are connected to lifestyle habits [16, 17]. Various psychosocial factors, such as motivation and self-discipline may be facilitating factors for lifestyle change; whereas feelings of uncertainty, lack of knowledge or enjoyment, and low mood can act as barriers [8, 18].

The Finnish Geriatric Intervention Study to Prevent Cognitive Impairment and Disability (FINGER) was the first large randomised controlled trial to show beneficial effects on cognitive performance utilising multidomain intervention [19]. Other large, but less intensive lifestyle-based trials have not confirmed these findings [20, 21], suggesting that adherence to intervention activities and lifestyle changes may play a crucial role in the success of lifestyle interventions [5]. Furthermore, the role of psychosocial factors for adherence to lifestyle changes or participation in lifestyle interventions is unclear, especially in the context of dementia prevention.

In this study, we aimed to examine whether psychosocial factors measured at trial baseline and earlier in life are associated with intervention participation, and with healthy lifestyle and lifestyle changes among the FINGER trial participants (exploratory analyses).

## Methods

### Study design and participants

The FINGER trial (ClinicalTrials.gov identifier NCT01041989) was a 2 year RCT conducted in six centres in Eastern Finland. Participants were recruited from earlier population-based observational surveys (the National FINRISK Study and the national type 2 diabetes prevention programme in Finland, FIN-D2D) as described earlier [19]. Inclusion criteria included age 60–77 years in the beginning of the study; elevated dementia risk identified with an established dementia risk score [22]; and cognitive performance at the mean level or slightly lower than expected for age.

**Fig. 1:**
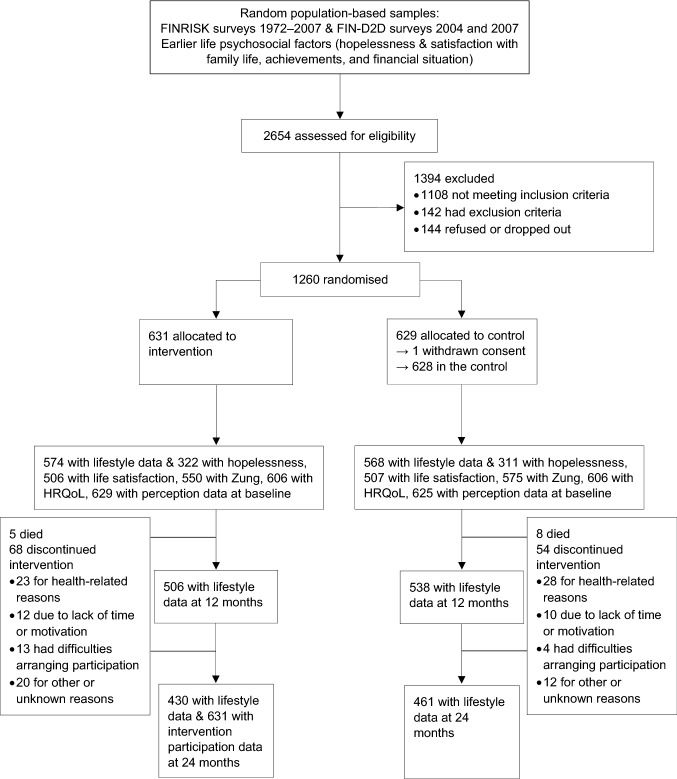
CONSORT flowchart of the study design. FINRISK denotes the National FINRISK study and FIN-D2D denotes the national type 2 diabetes prevention programme in Finland. *HRQoL*, health-related quality of life

The FINGER trial was approved by the Coordinating Ethics Committee of the Hospital District of Helsinki and Uusimaa and conducted following the Declaration of Helsinki and Good Clinical Practice. Written informed consent was received from every participant. Flow chart of the study design is provided as Fig. 1.

### Randomisation and masking

2654 individuals were screened for eligibility from September 7, 2009 to November 24, 2011, of whom 1260 were randomised in a 1:1 ratio to a multidomain lifestyle intervention or regular health advice (control) group (computer-generated randomisation was done in blocks of four individuals at each site). All participants received an oral mini-intervention at the beginning of the trial and regular feedback for their vascular risk factors during the trial. The intervention group received additionally dietary counselling, a physical exercise programme, cognitive training programme, and intensive management of vascular and metabolic risk factors for 2 years. The primary outcome was a change in global cognition [19]. The group allocation was not actively revealed to participants.

### Procedures

Interventions have been described previously [19]. In brief, dietary intervention included three individual counselling sessions and at least six group meetings with the study nutritionist. Individual sessions focused on individual goal setting and personal adjustments, while group sessions included discussions, group activities, and peer support. The goals of the dietary intervention were based on national recommendations, translated into food consumption targets [19].

The physical exercise intervention consisted of the physiotherapist-guided individually tailored progressive muscle strength training (1–3 times a week) that included also postural balance training, as well as independent aerobic exercise (2–5 times a week).

The cognitive training comprised six group sessions and an individualised computer-based training programme including sessions two to three times a week, totalling 144 sessions. Psychologist-led group sessions included educational discussions of age-related cognitive issues. Social activities were promoted during all group sessions and with a group visit to the local Alzheimer Association.

Management of vascular and metabolic risk factors was based on national guidelines and included individual counselling visits with the study nurse and physician, while getting feedback on their measured risk factors. If modifications of medical treatment were needed, participants were referred to their own primary health care.

### Psychosocial factors at FINGER baseline

Depressive symptoms were assessed with the Zung Self-Rating Depression Scale [23] containing 20 questions, of which half are positively and half negatively phrased. Four answer options are scored from 1 to 4 or 4 to 1, depending on the phrasing. A sum score ranges from 20 to 80 points, with a higher score representing more depressive symptoms. We allowed one missing value per participant, by replacing the missing value with a question-specific average value to calculate Zung total score.

*HRQoL* was assessed with the SF-36/RAND-36 questionnaire [24], comprising 36 questions divided into eight subscales: physical functioning, role limitations caused by physical health problems, role limitations caused by emotional problems, social functioning, emotional well-being, energy/fatigue, pain, and general health perceptions. In this study we used *HRQoL* mental and physical health component scores, derived from the eight subscales [25]. Both component scores range from 0 to 100, a higher score indicating better *HRQoL*.

Participants’ initial perception of the study was assessed by the question: “What is your perception of taking part in this lifestyle counselling study?” with answer options: (1) “Very positive”; (2) “Positive”; (3) “I can’t say”; (4) “Rather negative”; (5) “Very negative”; and (6) “Other, describe”. Answers were dichotomised into positive (1–2) and nonpositive (3–6), because a great majority of the participants (93%) had positive or very positive perception.

### Psychosocial factors from earlier life

Each FINGER participant had data from one observational background survey (FINRISK or FIN-D2D) carried out between 1972 and 2007.

Hopelessness was inquired only in the FINRISK at certain cohorts (1972–1997), with two statements “The future seems hopeless to me, and I don’t believe that things are changing for the better” and “I feel that it is impossible to reach the goals I would like to strive for”. Answers on these questions were combined into a hopelessness variable with a higher score reflecting more hopelessness, following earlier studies (range 0–8) [3].

Satisfaction with family life, achievements, and financial situation were evaluated in all earlier life questionnaires with one question on each. Answer options on these questions were provided on a five-point Likert scale ranging from “Very satisfied” to “Very dissatisfied”. We dichotomised answers to each question as satisfied versus dissatisfied, and those not having a family (separate option provided) were excluded from family life analyses.

### Intervention participation assessment

Participation in the dietary intervention included group and individual sessions; physical exercise intervention resistance training sessions and progress evaluation visits; cognitive training group sessions and completed computer training sessions; and cardiovascular disease (CVD) risk factor control visits (“CVD visit participation”). For each domain, 0 points were given for not active, 1 point for partially active (< 50%), and 2 points for active participants (50–100%) (pre-defined definition). These three-class variables were summed up for multidomain participation, ranging from 0 to 8 points. This was further categorised into low (0–5), intermediate (6–7), and high (8) multidomain participation. High participation was achieved by 88% in dietary intervention, 57% in exercise intervention, 47% in cognitive training, and 93% in CVD risk factor intervention. In total, 37% of participants reached high participation in all four domains.

### Lifestyle assessment

Lifestyle, including four separate domains and multidomain derived from these, was evaluated for both intervention and control groups at the FINGER trial baseline, 1 year, and 2 years. These annual study visits included anthropometric measurements, blood samples, and detailed questionnaires on background, lifestyle, and health.

Diet quality was assessed with three-day food records where participants recorded all foods and beverages consumed, to calculate a diet score reflecting adherence to national dietary recommendations and study goals (fish, fruit, and vegetable consumption; and intake of saturated fat, polyunsaturated fat, fibre, sucrose, protein, and alcohol), ranging 0–9.

Physical activity was measured with a self-reported questionnaire including frequencies of different sport modalities (walking, jogging, cross-country skiing, cycling, swimming, skating, rowing, golf, ball games, dancing, bowling, aerobics, gymnastics, gym exercises) over the past 12 months. An average number of moderate to vigorous sessions (lasting over 10 min) per week was calculated.

Cognitive and social activity was evaluated with a self-reported questionnaire including 12 activities (reading, crosswords, writing, games, listening/playing music, communal/society activities, studying, handicrafts, gardening, baby-sitting, voluntary work, and computer use). A total amount of activities per week was calculated.

CVD risk factor control was calculated based on the FINRISK risk score estimating the 10 year incidence of coronary heart disease and stroke. It comprises six established CVD risk factors: smoking, systolic blood pressure, total cholesterol, high-density lipoprotein cholesterol, diabetes, and family history [26]. The risk score was calculated in relation to age and sex by dividing absolute risk by reference risk, as described earlier [27], yielding a relative risk score per participant.

Each lifestyle domain was categorised into tertiles (for CVD risk control reversely), a higher class indicating healthier/more active lifestyle. Adherence to a multidomain healthy lifestyle was calculated as a continuous sum score from the aforementioned four lifestyle domain scores (tertiles scored from 0 to 2; multidomain score range 0–8), a higher score reflecting healthier lifestyle.

Change in each lifestyle domain was calculated as the difference between 2 years and baseline: participants were given 0 points for decline, 1 point for staying stable, and 2 points for improvement (thus, a higher class indicating more favourable change). This determination was based on estimated clinical sensibility and variation in the population (for diet change of ≥ 1 point on the 9-item score; for physical activity change of > 2 sessions/week; for social/cognitive activities change of > 3 activities/week; and for CVD risk control change of > 0.5%). Change in multidomain lifestyle was assessed using the continuous 8-point score measured at three time points (baseline, 1 year, and 2 years).

### Covariates

Sociodemographic factors (age, sex, education, income, and marital status) were collected from population registers or by questionnaires at FINGER baseline and earlier FINRISK/FIN-D2D visits. Marital status was divided into married/cohabiting vs other. Antidepressant use was self-reported and confirmed with the study physician at FINGER screening and 2 years, and was further combined into antidepressant use at either time point vs no antidepressant use. Information on earlier life diagnosed depression, self-reported depressive symptoms, and self-reported antidepressant medication use was collected from FINRISK/FIN-D2D surveys, and these were combined into an indicator of depression experienced earlier in life as having at least one sign of depression or none.

### Statistical analyses

We compared background characteristics between the randomisation groups and between those with and without missing data using *χ*^2^ test, *t* test, or Mann–Whitney *U* test, as appropriate.

Continuous multidomain lifestyle index was analysed with linear regression (cross-sectional associations with baseline psychosocial variables and longitudinal analyses with earlier life variables in relation to baseline lifestyle) and with mixed-effects repeated-measures regression (longitudinal associations regarding lifestyle change over the two-year period using three time points).

Categorised multidomain participation, and categorical domain-specific participation and lifestyle variables (diet, physical activity, social/cognitive activity, and CVD risk control) were analysed with generalised ordinal logistic regression (cross-sectional associations with baseline psychosocial factors, longitudinal associations with earlier life psychosocial factors, and longitudinal associations of lifestyle changes and participation with psychosocial factors) because some of the analyses did not fulfil the proportional odds assumption of traditional ordinal logistic regression.

In the mixed-effects model, we included psychosocial factor × time interaction to evaluate associations of each factor with lifestyle change (intervention and control groups combined) and factor × time × group interaction to evaluate the possible modification of intervention effect by psychosocial factors. To improve interpretation, we further dichotomised psychosocial variables (continuous based on a median value) to determine estimates for the intervention effect within psychosocial factor subgroups (group × time × dichotomised factor with Lincom postestimation command in Stata).

Analyses including baseline psychosocial factors were adjusted for age, sex, education, marriage/cohabiting, and trial site at baseline, and use of antidepressants at screening or ever. Analyses using FINRISK/FIN-D2D psychosocial factors were adjusted for marriage/cohabiting and signs of depression in earlier life, follow-up time from earlier life survey to FINGER screening, age in earlier life (for baseline analyses) or at baseline (for analyses of the trial period), and sex, education, and trial site at baseline. Mixed model analyses evaluating change in multidomain lifestyle among the entire population were additionally adjusted for group × time interaction. All analyses were conducted with Stata software version 14 for Windows (StataCorp.). The level of statistical significance was < 0.05.

## Results

We had data on 1259 randomised participants, with a mean age of 69 years at baseline and 56 years at the previous surveys. There were no differences in the FINGER baseline characteristics between the randomisation groups (Online Resource 1), but more people in the intervention group were dissatisfied with their financial situation in the earlier survey.

Baseline multidomain lifestyle score was not calculated for 117 persons (9.3%), because of missing values: 6 for diet, 82 for physical activity, 5 for social/cognitive activities, and 27 for CVD risk estimate. People with missing data were more often dissatisfied with their achievements in earlier surveys (*P* = 0.019, results not shown), but there were no other differences compared with included participants.

### Psychosocial factors and intervention participation

Table 1 shows that more hopelessness earlier in life was associated with poorer overall participation, particularly poorer cognitive training participation. No other psychosocial factors from earlier life were related to participation, whereas all investigated baseline factors were. Higher depressive symptoms, nonpositive study perception, and lower *HRQoL* mental and physical components were associated with poorer overall participation. All of these were significantly associated with some of the individual domains, but none were related to CVD risk factor control visit participation.

**Table 1:** Associations of participation in the intervention with psychosocial factors earlier in life and at baseline among the FINGER intervention group participants

	Overall participation	Diet participation	Exercise participation	Cognitive training participation	CVD risk factor control visit participation
Psychosocial factors from FINRISK/FIN-D2D surveys^a^					
	Coefficient (95% CI)				
Hopelessness	**− 0.15 (− 0.29** to 0.018)*	− 0.13 (− 0.35 to 0.10)	− 0.087 (− 0.23 to 0.052)	**− 0.14 (− 0.29** to 0.003)*	− 0.11 (− 0.36 to 0.14)
Dissatisfaction with family life	− 0.15 (− 0.93 to 0.63)	− 0.038 (− 1.31 to 1.24)	− 0.29 (− 1.12 to 0.55)	− 0.13 (− 0.94 to 0.68)	0.55 (− 1.51 to 2.61)
Dissatisfaction with achievements	0.44 (− 0.16 to 1.04)	0.76 (− 0.49 to 2.00)	0.43 (− 0.23 to 1.08)	0.29 (− 0.33 to 0.91)	1.36 (− 0.68 to 3.40)
Dissatisfaction with financial situation	− 0.014 (− 0.52 to 0.49)	0.18 (− 0.69 to 1.05)	− 0.017 (− 0.55 to 0.51)	− 0.11 (− 0.61 to 0.39)	0.58 (− 0.66 to 1.81)
Psychosocial factors at FINGER baseline^b^					
	Coefficient (95% CI)				
Depressive symptoms (Zung)	**− 0.031 (− 0.052** to 0.010)**	− 0.017 (− 0.051 to 0.018)	**− 0.031 (− 0.052** to 0.009)**	− 0.021 (− 0.042 to 0.0004)	0.002 (− 0.043 to 0.046)
*HRQoL* mental component	**0.023 (0.005** to 0.041)*	0.010 (− 0.20 to 0.040)	0.012 (− 0.006 to 0.031)	**0.019 (0.001** to 0.038)*	− 0.011 (− 0.051 to 0.030)
*HRQoL* physical component	**0.018 (0.001** to 0.035)*	0.016 (− 0.012 to 0.044)	0.002 (− 0.024 to 0.029)^c^ **0.033 (0.014** to 0.052)**^d^	0.010 (− 0.007 to 0.028)	0.024 (− 0.010 to 0.059)
Nonpositive perception of the study	**− 0.90 (− 1.50** to 0.30)**	**− 0.82 (− 1.60** to 0.043)*	**− 0.90 (− 1.50** to 0.30)**	**− 0.76 (− 1.35** to 0.17)**	− 0.76 (− 1.72 to 0.20)

^a^Generalised ordinal logistic regression analyses adjusted for age at baseline, sex, education, marriage/cohabiting status in earlier life survey, study site, follow-up time, and signs of depression (depressive symptoms and/or use of antidepressants) in earlier life survey

^b^Generalised ordinal logistic regression analyses adjusted for age at baseline, sex, education, marriage/cohabiting status at baseline, study site, and use of antidepressants. Two different coefficients refer to that there are nonparallel associations between predictor variable and separate ordinal outcome categories

^c^Active/partially active vs not active category

^d^Active vs partially active/not active category

FINRISK denotes the National FINRISK study and FIN-D2D denotes the national type 2 diabetes prevention programme in Finland. *HRQoL*, health-related quality of life. *P* values < 0.05 are in bold. *P < 0.05 **P < 0.01 ***P < 0.001

### Psychosocial factors and lifestyle at baseline

Psychosocial factors from both earlier life and baseline showed association with baseline lifestyle among the entire trial population (Table 2). More hopelessness and dissatisfaction with financial situation earlier in life predicted unhealthier multidomain lifestyle, especially lower level of physical and social/cognitive activities. Dissatisfaction with family life was associated with unhealthier baseline diet, and dissatisfaction with achievements to less social/cognitive activities. A higher baseline depressive symptom score and lower *HRQoL* physical component were cross-sectionally associated with unhealthier multidomain lifestyle. As for the individual domains, more pronounced depressive symptoms and lower *HRQoL* mental component were associated with unhealthier diet and less social/cognitive activities, whereas higher *HRQoL* physical component was associated with physical activity, and higher physical but lower mental *HRQoL* component to better CVD risk factor control.

**Table 2:** Associations of baseline lifestyle with psychosocial factors earlier in life and at baseline

	Multidomain healthy lifestyle^a^	Healthy diet^b^	Physical activity^b^	Social/cognitive activities^b^	CVD risk factor control^b^
Psychosocial factors from FINRISK/FIN-D2D surveys					
	Coefficient (95% CI)				
Hopelessness	**− 0.17 (− 0.25** to 0.085)***	− 0.070 (− 0.17 to 0.032)	**− 0.13 (− 0.22** to 0.031)**	**− 0.18 (− 0.29** to 0.076)**^c^ − 0.033 (− 0.14 to 0.073)^d^	− 0.088 (− 0.18 to 0.005)
Dissatisfaction with family life	− 0.24 (− 0.76 to 0.27)	**− 0.76 (− 1.38** to 0.14)*	0.14 (− 0.45 to 0.74)	− 0.45 (− 1.04 to 0.14)	0.52 (− 0.049 to 1.08)
Dissatisfaction with achievements	− 0.34 (− 0.73 to 0.058)	− 0.26 (− 0.75 to 0.22)	− 0.33 (− 0.80 to 0.13)	**− 0.48 (− 0.96** to 0.004)*^c^ − 0.013 (− 0.53 to 0.50)^d^	− 0.16 (− 0.61 to 0.28)
Dissatisfaction with financial situation	**− 0.40 (− 0.73** to 0.060)*	− 0.24 (− 0.65 to 0.18)	**− 0.78 (− 1.22** to 0.35)***^c^ − 0.082 (− 0.53 to 0.36)^d^	**− 0.41 (− 0.82** to 0.0005)*^c^ 0.21 (− 0.23 to 0.64)^d^	− 0.22 (− 0.61 to 0.16)
Psychosocial factors at FINGER baseline					
	Coefficient (95% CI)				
Depressive symptoms (Zung)	**− 0.020 (− 0.033** to 0.007)**	**− 0.021 (− 0.038** to 0.005)*	− 0.013 (− 0.028 to 0.003)	**− 0.015 (− 0.030** to 0.0004)*	− 0.016 (− 0.032 to 0.001)^c^ 0.005 (− 0.013 to 0.022)^d^
*HRQoL* mental component	0.008 (− 0.003 to 0.019)	**0.024 (0.011** to 0.038)***	0.0003 (− 0.012 to 0.013)	**0.014 (0.002** to 0.026)*	− 0.004 (− 0.018 to 0.010)^c^ **− 0.019 (− 0.033** to 0.005)*^d^
*HRQoL* physical component	**0.022 (0.012** to 0.032)***	− 0.003 (− 0.017 to 0.010)	**0.033 (0.020** to 0.047)***^c^ **0.018 (0.003** to 0.032)*^d^	0.003 (− 0.009 to 0.015)	**0.038 (0.024** to 0.052)***^c^ **0.015 (0.0008** to 0.029)*^d^
Nonpositive perception of the study	− 0.29 (− 0.66 to 0.072)	− 0.36 (− 0.82 to 0.11)	− 0.18 (− 0.61 to 0.25)	− 0.11 (− 0.52 to 0.31)	− 0.19 (− 0.62 to 0.25)

^a^Linear regression adjusted for age in earlier life survey, sex, education, marriage/cohabiting status in earlier life survey, study site, follow-up time, and signs of depression (depressive symptoms and/or use of antidepressants) in earlier life survey for FINRISK/FIN-D2D factors; linear regression adjusted for age at baseline, sex, education, marriage/cohabiting status at baseline, study site, and use of antidepressants at screening for FINGER factors

^b^Generalized ordinal logistic regression adjusted for age in earlier life survey, sex, education, marriage/cohabiting status in earlier life survey, study site, follow-up time, and signs of depression (depressive symptoms and/or use of antidepressants) in earlier life survey for FINRISK/FIN-D2D factors; generalised ordinal logistic regression adjusted for age at baseline, sex, education, marriage/cohabiting status at baseline, study site, and use of antidepressants at screening for FINGER factors. Two different coefficients refer to that there are nonparallel associations between predictor variable and separate ordinal outcome categories

^c^High/intermediate vs low category

^d^High vs intermediate/low category

FINRISK denotes the National FINRISK study and FIN-D2D denotes the national type 2 diabetes prevention programme in Finland. *HRQoL*, health-related quality of life. *P*-values < 0.05 are in bold. *P < 0.05 **P < 0.01 ***P < 0.001

### Psychosocial factors and lifestyle changes

Table 3 shows that psychosocial factors earlier in life were unrelated to lifestyle change during the 2 years, but higher baseline depressive symptom score and lower *HRQoL* physical component were associated with unhealthier lifestyle change. In the analyses stratified by intervention allocation, depressive symptoms were associated with unhealthier lifestyle change specifically in the intervention group (1st year estimate [95% CI] − 0.016 [− 0.033 to − 0.00004]; 2nd year − 0.021 [− 0.039 to − 0.004]), but *HRQoL* physical component prominently in the control group (1st year 0.017 [0.003 to 0.030]; 2nd year 0.022 [0.008 to 0.036]).

**Table 3:** Associations of multidomain lifestyle change with psychosocial factors earlier in life and at baseline

	Estimate (95% CI) for the association of psychosocial factor with change in lifestyle	
	Year 1	Year 2
Psychosocial factors from FINRISK/FIN-D2D surveys^a^		
Hopelessness	− 0.041 (− 0.11 to 0.031)	− 0.050 (− 0.12 to 0.023)
Dissatisfaction with family life	0.088 (− 0.38 to 0.55)	0.28 (− 0.22 to 0.78)
Dissatisfaction with achievements	− 0.059 (− 0.42 to 0.30)	− 0.15 (− 0.53 to 0.24)
Dissatisfaction with financial situation	0.17 (− 0.14 to 0.49)	0.19 (− 0.14 to 0.51)
Psychosocial factors at FINGER baseline^b^		
Depressive symptoms (Zung)	− 0.011 (− 0.022 to 0.0007)	− **0.018 (**− **0.030** to 0.006)**
*HRQoL* mental component	0.004 (− 0.006 to 0.014)	0.009 (− 0.002 to 0.019)
*HRQoL* physical component	**0.011 (0.002** to 0.021)*	**0.016 (0.006** to 0.027)**
Nonpositive perception of the study	0.077 (− 0.28 to 0.43)	− 0.013 (− 0.39 to 0.37)

^a^Mixed-effects regression analyses adjusted for age at baseline, sex, education, marriage/cohabiting status in earlier life survey, study site, follow-up time, signs of depression (depressive symptoms and/or use of antidepressants) in earlier life survey, and allocation group × time interaction

^b^Mixed-effects regression analyses adjusted for age at baseline, sex, education, marriage/cohabiting status at baseline, study site, use of antidepressants, and allocation group × time interaction

FINRISK denotes the National FINRISK study and FIN-D2D denotes the national type 2 diabetes prevention programme in Finland. *HRQoL*, health-related quality of life. *P* values < 0.05 are in bold. *P < 0.05 **P < 0.01 ***P < 0.001

Table 4 shows that there were few interactions between psychosocial factors and group allocation for a multidomain lifestyle change, suggesting that the intervention was beneficial for lifestyle change independent of most studied psychosocial characteristics. This is indicated by positive estimates for the difference between the randomisation groups. The only significant interaction observed was for dissatisfaction with achievements, with dissatisfied participants benefitting more from the intervention particularly during the first year. Dissatisfaction with financial situation in earlier life tended to be also related to more benefits of the intervention (Table 4). Adjustment for baseline income did not significantly change these results (results not shown).

**Table 4:** Intervention effect on multidomain lifestyle change according to psychosocial factor subgroups earlier in life and at baseline

Dichotomised baseline factor (*N* intervention/*N* control)		Estimate (95% CI) for the difference between the intervention and control groups		Estimate (*P*) for factor × group × time interaction§	
		Year 1	Year 2	Year 1	Year 2
Psychosocial factors from FINRISK/FIN-D2D surveys^a^					
Hopelessness	< 3 (159/166)	**0.40 (0.056** to 0.74)*	0.19 (− 0.16 to 0.54)	0.003 (0.97)	0.033 (0.66)
	≥ 3 (163/145)	0.31 (− 0.037 to 0.66)	0.25 (− 0.10 to 0.61)		
Dissatisfaction with family life	No (485/490)	**0.22 (0.025** to 0.41)*	**0.23 (0.025** to 0.43)*	− 0.22 (0.64)	0.049 (0.92)
	Yes (25/19)	− 0.004 (− 0.92 to 0.92)	0.28 (− 0.71 to 1.27)		
Dissatisfaction with achievements	No (546/546)	**0.21 (0.030** to 0.39)*	0.19 (− 0.003 to 0.38)	0.83 (0.025)*	0.72 (0.068)
	Yes (45/39)	**1.04 (0.34** to 1.75)**	**0.91 (0.16** to 1.65)*		
Dissatisfaction with financial situation	No (519/543)	**0.21 (0.026** to 0.39)*	0.19 (− 0.006 to 0.38)	0.58 (0.071)	0.64 (0.056)
	Yes (69/45)	**0.79 (0.19** to 1.39)*	**0.83 (0.20** to 1.46)*		
Psychosocial factors at FINGER baseline^b^					
Depressive symptoms (Zung)	< 33 (286/279)	**0.32 (0.074** to 0.57)*	**0.32 (0.056** to 0.58)*	− 0.011 (0.34)	− 0.006 (0.62)
	≥ 33 (320/338)	0.19 (− 0.045 to 0.43)	0.22 (− 0.030 to 0.47)		
*HRQoL* mental component	< 57 (299/310)	**0.27 (0.028** to 0.51)*	**0.39 (0.13** to 0.65)**	0.002 (0.86)	− 0.009 (0.42)
	≥ 57 (307/296)	**0.25 (0.010** to 0.49)*	0.15 (− 0.11 to 0.40)		
*HRQoL* physical component	< 49 (327/305)	**0.31 (0.067** to 0.54)*	**0.38 (0.12** to 0.63)**	− 0.011 (0.25)	− 0.012 (0.24)
	≥ 49 (279/301)	0.22 (− 0.021 to 0.47)	0.18 (− 0.081 to 0.43)		
Nonpositive perception of the study	No (584/586)	**0.24 (0.065** to 0.41)**	**0.30 (0.12** to 0.49)**	0.54 (0.14)	− 0.52 (0.18)
	Yes (45/39)	**0.78 (0.093** to 1.46)*	− 0.21 (− 0.95 to 0.52)		

Mixed-effects regression models with repeated measures were used to analyse whether the studied psychosocial factors affected intervention benefits on multidomain lifestyle change (factor × group × time interaction). Original continuous or categorical variables were recoded, when needed (if not already dichotomy), into dichotomous variables (continuous based on a median value) to determine estimates for the differences between the intervention and control groups within subgroups per year (group × time × dichotomised factor with Lincom postestimation command in Stata). A positive value of the estimate for the difference between the intervention and control groups indicates that lifestyle change within the subgroup is in favour of the intervention group. Data are based on participants with at least one lifestyle assessment

^a^Analyses are adjusted for age at baseline, sex, education, marriage/cohabiting status in earlier life survey, study site, follow-up time, and signs of depression (depressive symptoms and/or use of antidepressants) in earlier life survey

^b^Analyses are adjusted for age at baseline, sex, education, marriage/cohabiting status at baseline, study site, and use of antidepressants

^c^Estimate (*P*) for the three-way interaction is presented for the whole group within each categorised or continuous factor (when not dichotomised into subgroups)

FINRISK denotes the National FINRISK study and FIN-D2D denotes the national type 2 diabetes prevention programme in Finland. *HRQoL*, health-related quality of life. *P*-values < 0.05 are in bold. *P < 0.05 **P < 0.01 ***P < 0.001

In line with associations between psychosocial factors and multidomain lifestyle change, some associations were found for change in individual domains (Online Resource 2). Depressive symptoms were associated with less improvement in physical activity and CVD risk factor control, whereas higher *HRQoL* physical component was associated with healthier change in diet, social/cognitive activities, and CVD risk factor control among the entire population (nonsignificant factor × group interactions). Nonpositive study perception was associated with an increase in social/cognitive activities, more prominently in the control group (estimate [95% CI] was 1.23 [0.45 to 2.01]) than in the intervention group (0.12 [− 0.54 to 0.77]), with significant factor × group interaction (*P* = 0.027).

## Discussion

In the FINGER multidomain lifestyle trial among older people, we found that the intervention was beneficial for lifestyle change independent of most studied psychosocial factors. All psychosocial factors assessed at baseline were, however, associated with participation in the intervention. While psychosocial factors from both earlier in life and at baseline were associated with baseline lifestyle, lifestyle change during the trial was particularly related to baseline psychosocial factors. Associations of psychosocial factors with lifestyle change and participation were conveyed via several lifestyle domains.

Our results showed that depressive symptoms, *HRQoL*, initial study perception, and earlier life hopelessness affected participation in the intervention. Earlier studies regarding effects of depressive symptoms on participation have yielded somewhat inconsistent results [5, 7, 9]. Dichotomised depression measure was associated with participation in exercise intervention in the previous analysis of the FINGER [5]. This study used a different definition for adherence in order to harmonise results between two trials, whereas we used the pre-specified definition and found association also with overall participation. In addition, the nonpositive perception was significant predictor of cognitive training participation in the previous report [5], whereas we observed its negative association with participation in several intervention domains. We could not identify other publications that included the study perception, but this concept has been suggested to be used as a surrogate for self-efficacy that has been previously connected to exercise participation [11, 12], likewise has a positive attitude [6]. In addition, earlier study focusing on computer-based cognitive training in the FINGER found that positive expectations were associated with joining the programme [28].

Another previous FINGER-based analysis showed that the multidomain lifestyle intervention improved some dimensions of *HRQoL* [29]. Regarding the predictive role of *HRQoL*, in line with our findings of the *HRQoL* physical component, a study with lifestyle intervention among middle-aged obese adults found that low *HRQoL* was associated with poorer participation in exercise sessions [7]. A slightly different pattern was observed in a small physical activity study among stroke survivors, where a better quality of life was associated with more independent exercise measured with a pedometer, but not with participation in group exercise sessions [30]. As far as we know, hopelessness has not previously been studied in relation to intervention participation.

In the intervention settings, determinants of lifestyle change are sometimes conceptually hard to separate from determinants of participation, although active participation does not automatically predict successful lifestyle change. It is thus worth studying whether predictors for these differ. We found associations of depressive symptoms and the *HRQoL* physical component with lifestyle change during the trial. Our findings are in line with previous research suggesting that low mood can be a barrier to lifestyle change [8], whereas quality of life may facilitate lifestyle change [13]. Lower participation in the intervention may be one factor mediating the association between depressive symptoms and unhealthier lifestyle change, and the fact that this effect on lifestyle change was seen especially in the intervention group supports this mechanism. Depressive symptoms may also hamper individuals from taking care of themselves, which may explain the associations of depressive symptoms and negative changes in CVD risk factor control and physical activity seen in this study.

The *HRQoL* physical component was connected to healthier changes in diet, social/cognitive activities, and CVD risk management, suggesting that physically healthier people may have better resources to make changes on several metabolic risk factors, as well as healthier lifestyles that contribute to these risk factors. The association between the *HRQoL* physical component and healthier lifestyle at baseline supports this assumption. The *HRQoL* physical component was rather expectedly linked to higher exercise participation, which may have been reflected in beneficial lifestyle changes. However, the association with a multidomain lifestyle change was predominant among the control group participants. It should be noted that also control group received mini-intervention and health advice, and their CVD risk factor levels were followed rather carefully during the trial. After feedback, maybe only the control participants who were physically capable to make changes attempted to change, whereas all intervention group participants had a rather intensive programme despite their initial physical health.

The associations of earlier life dissatisfaction with achievements and financial situation with a healthier lifestyle change favouring intervention group may reflect that these people took a good advantage of the offered intervention. The results were not, however, explained by the actual financial situation at baseline, nor was dissatisfaction associated with intervention participation. It is also noticeable that there was room for improvement because aspects of dissatisfaction were associated with baseline unhealthier lifestyle habits. We found no previous studies examining life satisfaction in relation to a lifestyle change. Interestingly, the nonpositive perception was associated with a positive change in social/cognitive activities in the control group. Participants in the intervention group may not have had the time or need to increase their leisure-time activities due to otherwise activating and frequent intervention, but it is unclear why this activity increase in the control group was seen in people with nonpositive perception.

Our results also showed that various psychosocial factors were associated with lifestyle habits already at baseline. Depressive symptoms have been consistently connected to an unhealthier lifestyle also previously [14, 15]. Thus, depressive symptoms seem important to affect habitual lifestyle, along with hindering individuals from engaging actively in intensive lifestyle modification, especially physical exercise. The *HRQoL* mental component may have an association with to depressive symptomatology, but it reflects mental health in a reverse, positive manner. Indeed, in our sample it correlated inversely with the depressive symptom score, and its associations with baseline diet and social/cognitive activities were inversely comparable to those of depressive symptoms. Mental health is, however, not a direct inverse of depressive symptoms, and some findings differed between these factors. The *HRQoL* mental component may include more aspects connected to cognitive abilities and, accordingly, this factor was associated with cognitive training participation. Also the physical component of *HRQoL* was associated with many aspects of baseline lifestyle, suggesting that physical well-being enables to pursue and maintain healthy lifestyle habits.

To the best of our knowledge, our study was the first one showing that a history of hopelessness and dissatisfaction with important life aspects were associated with lifestyle habits. Hopelessness and dissatisfaction may reduce motivation to focus on a healthy lifestyle, or be an indicator of other problems acting as barriers to following a healthy lifestyle. These findings reflect the multiple and long-term consequences of earlier life dissatisfaction and hopelessness. Previously, midlife hopelessness has been found to be associated with the development of cognitive impairment and Alzheimer’s disease [3].

### Strengths and limitations

The main strengths of this study include a large population-based sample, well-defined population, low drop-out rate, and versatile psychosocial and lifestyle measurements. We were able to consider complementary psychosocial factors from both earlier life and trial baseline and study their role for both lifestyle change and intervention participation. Furthermore, our outcomes included multidomain variables in addition to separate lifestyle/participation domains, which is rare in previous studies. Limitations of this study include that the multidomain lifestyle variable was derived from four domains to meet the FINGER intervention domains, and does not include all aspects of a healthy lifestyle. In addition, the definition of the lifestyle changes was based on rather arbitrary cut-offs that may not be sensitive to capture smaller changes. Furthermore, participation in the trial may have affected the reporting of lifestyle habits, which may have confounded the results. Individuals with major depression were excluded from the trial, and it is possible that individuals with extreme answers on other psychosocial questionnaires are less likely to participate in trials in general. However, it is noteworthy that several psychosocial factors were related to participation and lifestyle also among people with relatively mild psychological problems. Also, excluded people could have been the ones with the most extreme lifestyle habits, and their exclusion is more likely to underestimate than overestimate the results presented. It is also noteworthy that hopelessness questions were not included in all earlier life surveys, leading to a smaller population, and answers indicating nonpositive perception and dissatisfaction were quite rare. These matters may have led to a limited power to detect significant associations. Furthermore, we want to point out that the analyses presented in this paper were exploratory in nature.

To conclude, the FINGER lifestyle intervention was largely beneficial for lifestyle change independent of psychosocial factors present before and during the trial. However, several factors were associated with participation in the intervention and lifestyle habits, and some also with lifestyle change during the trial. Future lifestyle-modifying trials should consider including psychosocial intervention domains or tailored psychosocial support for individuals with the most vulnerable psychosocial profiles.

## Supplementary Information

Below is the link to the electronic supplementary material.

Supplementary file1 (DOCX 34 KB)

## Data Availability

Data used in this study are not publicly available due to ethical and legal reasons, but data are available upon request. Those fulfilling the requirements for viewing confidential data as required by the Finnish legislation and the Finnish Institute for Health and Welfare are able to access the data after completion of material transfer agreement. Requests may be directed to kirjaamo@thl.fi.
