# Supplementary material for: Psychosocial determinants for adherence to a healthy lifestyle and intervention participation in the FINGER trial: an exploratory analysis of a randomised clinical trial
Source: Aging Clin Exp Res. 2022 Feb 19;34(8):1793–805. doi: 10.1007/s40520-022-02088-x (PMC9283154; doi:10.1007/s40520-022-02088-x)
Supplement: Supplementary file 1 — Supplementary file1 (DOCX 34 KB) [file 40520_2022_2088_MOESM1_ESM.docx]

**Supplementary Information**

**Psychosocial determinants for adherence to a healthy lifestyle and intervention participation in the FINGER trial: an exploratory analysis of a randomised clinical trial**

**Aging Clinical and Experimental Research**

**Elisa Neuvonen, MD, Jenni Lehtisalo, PhD, Alina Solomon, MD, PhD, Riitta Antikainen, MD, PhD, Satu Havulinna, PhD, Tuomo Hänninen, PhD, Tiina Laatikainen, MD, PhD, Jaana Lindström, PhD, Nina Rautio PhD, Hilkka Soininen, MD, PhD, Timo Strandberg, MD, PhD, Jaakko Tuomilehto, MD, PhD, Miia Kivipelto, MD, PhD, Tiia Ngandu, MD, PhD**

**Correspondence to Elisa Neuvonen; University of Eastern Finland, School of Medicine; Email** [**elisa.neuvonen@uef.fi**](mailto:elisa.neuvonen@uef.fi)

**Online Resource 1: Characteristic comparison earlier in life and at baseline according to allocation group**

|  | | **N (data**  **analysed)** | **Mean (SD) or n (%)** | | **p** |
| --- | --- | --- | --- | --- | --- |
|  |  |  | **Intervention** | **Control** |  |
| **From FINRISK/FIN-D2D surveys** | | | | | |
| Age (years) | | 1257 | 56.2 (10.9) | 56.1 (10.6) | 0.70 |
| Married/cohabiting | | 1183 | 461 (77.5%) | 469 (79.8%) | 0.34 |
| Signs of depression | | 1174 | 213 (36.1%) | 230 (39.4%) | 0.25 |
| Follow-up time from earlier life surveys to FINGER (years) | | 1259 | 13.4 (10.1) | 13.2 (10.1) | 0.63 |
| Hopelessness | | 633 | 2.6 (1.7) | 2.6 (1.8) | 0.50 |
| Dissatisfied with family life | | 1019 | 25 (4.9%) | 19 (3.7%) | 0.36 |
| Dissatisfied with achievements | | 1176 | 45 (7.6%) | 39 (6.7%) | 0.53 |
| Dissatisfied with financial situation | | 1176 | 69 (11.7%) | 45 (7.7%) | **0.018** |
| **At FINGER baseline** | | | | | |
| Age (years) | | 1259 | 69.5 (4.7) | 69.2 (4.7) | 0.24 |
| Sex: women | | 1259 | 286 (45.3%) | 301 (47.9%) | 0.35 |
| Education (years) | | 1257 | 10.0 (3.5) | 10.0 (3.4) | 0.98 |
| Married/cohabiting | | 1252 | 459 (73.3%) | 473 (75.6%) | 0.36 |
| Antidepressant use | | 1259 | 36 (5.7%) | 37 (5.9%) | 0.89 |
| Zung score | | 1223 | 34.0 (7.7) | 34.0 (7.2) | 0.71 |
| HRQoL mental component | | 1212 | 54.5 (8.9) | 54.3 (9.0) | 0.59 |
| HRQoL physical component | | 1212 | 45.7 (9.4) | 46.6 (8.9) | 0.13 |
| Nonpositive perception of the study | | 1254 | 45 (7.2%) | 39 (6.2%) | 0.52 |
| Diet score | 0 | 1252 | 100 (15.9%) | 110 (17.6%) | 0.71 |
|  | 1 |  | 411 (65.6%) | 405 (64.8%) |  |
|  | 2 |  | 116 (18.5%) | 110 (17.6%) |  |
| Physical activity score | 0 | 1176 | 208 (35.3%) | 183 (31.2%) | 0.22 |
|  | 1 |  | 185 (31.4%) | 209 (35.6%) |  |
|  | 2 |  | 196 (33.3%) | 195 (33.2%) |  |
| Social/cognitive activity score | 0 | 1254 | 218 (34.7%) | 197 (31.5%) | 0.42 |
|  | 1 |  | 211 (33.6%) | 211 (33.8%) |  |
|  | 2 |  | 200 (31.8%) | 217 (34.7%) |  |
| CVD risk control score | 0 | 1232 | 213 (34.4%) | 197 (32.1%) | 0.52 |
|  | 1 |  | 209 (33.8%) | 203 (33.1%) |  |
|  | 2 |  | 197 (31.8%) | 213 (34.8%) |  |
| Multidomain lifestyle score | | 1142 | 4.0 (1.6) | 4.1 (1.6) | 0.11 |

Values are reported as means (SD) for continuous variables, and as n (%) for categorical variables. FINRISK denotes the National FINRISK study and FIN-D2D denotes the national type 2 diabetes prevention programme in Finland. HRQoL, health-related quality of life.

**Online Resource 2: Earlier life and baseline psychosocial factors in relation to change in lifestyle domains**

|  | **Healthy diet** | **Physical activity** | **Social/cognitive activities** | **CVD risk factor control** |
| --- | --- | --- | --- | --- |
| **Psychosocial factors from**  **FINRISK/FIN-D2D surveys**† | | | | |
|  | **Coefficient (95% CI)** | | | |
| Hopelessness | -0.050 (-0.15-0.047) | 0.017 (-0.092-0.13) | -0.050 (-0.15-0.054) | -0.091 (-0.20-0.014) |
| Dissatisfaction with family life | 0.38 (-0.34-1.10)^§^  -0.64 (-1.38-0.097)^¶^ | 0.26 (-0.43-0.96) | 0.13 (-0.49-0.76) | -0.16 (-0.78-0.47) |
| Dissatisfaction with achievements | -0.20 (-0.69-0.28) | 0.30 (-0.25-0.85) | 0.029 (-0.47-0.52) | 0.013 (-0.49-0.51) |
| Dissatisfaction with financial situation | -0.037 (-0.45-0.37) | 0.25 (-0.21-0.71) | 0.26 (-0.16-0.68) | 0.21 (-0.21-0.63) |
| **Psychosocial factors at**  **FINGER baseline**‡ | | | | |
|  | **Coefficient (95% CI)** | | | |
| Depressive symptoms (Zung) | -0.008 (-0.024-0.009) | 0.002 (-0.021-0.026)^§^  **-0.025 (-0.046- -0.004)***^¶^ | -0.011 (-0.028-0.005) | **-0.033 (-0.050- -0.017)***** |
| HRQoL mental component | -0.005 (-0.018-0.009) | 0.011 (-0.004-0.026) | 0.003 (-0.010-0.017) | 0.010 (-0.004-0.024) |
| HRQoL physical component | **0.015 (0.001-0.028)*** | 0.007 (-0.007-0.022) | **0.021 (0.005-0.037)***^§^  0.0002 (-0.015-0.015)^¶^ | **0.022 (0.008-0.036)**** |
| Nonpositive perception of the study | -0.18 (-0.67-0.31) | -0.016 (-0.53-0.50) | **0.56 (0.066-1.06)*** | -0.15 (-0.65-0.35) |

FINRISK denotes the National FINRISK study and FIN-D2D denotes the national type 2 diabetes prevention programme in Finland. HRQoL, health-related quality of life. P-values <0.05 are in bold. *P<0.05 **P<0.01 ***P<0.001

† Generalized ordinal logistic regression analyses among the entire population with both the intervention and control group participants, adjusted for age at baseline, sex, education, marriage/cohabiting status in earlier life survey, study site, follow-up time, signs of depression (depressive symptoms and/or use of antidepressants) in earlier life survey, and group allocation. Two different coefficients refer to that there are nonparallel associations between predictor variable and separate ordinal outcome categories.

‡ Generalized ordinal logistic regression analyses among the entire population with both the intervention and control group participants, adjusted for age at baseline, sex, education, marriage/cohabiting status at baseline, study site, use of antidepressants, and group allocation. Two different coefficients refer to that there are nonparallel associations between predictor variable and separate ordinal outcome categories.

§ High/intermediate vs low category

¶ High vs intermediate/low category
